# Diagnosis and hormonal treatment of cystic ovaries associated with uterine disorders in Egyptian buffaloes: ultrasonography, histopathological and serological investigations

**DOI:** 10.1007/s11250-024-04220-7

**Published:** 2024-11-04

**Authors:** Mervat S. Hassan, Madeha Ahmed Hashim, Hayat Fayed, Fatma Abo Zakaib Ali

**Affiliations:** 1 Department of Theriogenology, Faculty of Veterinary Medicine, The New-Valley University, New Valley, 725211 Egypt; 2 Department of Histology, Faculty of Veterinary Medicine, Sohag University, P.O. Box 82524, Sohag, Egypt; 3 Department of Animal Medicine, Faculty of Veterinary Medicine, Benha University, P.O. Box 13511, Benha, Egypt; 4 Department of Pathology and Clinical Pathology, Faculty of Veterinary Medicine, Sohag University, P.O. Box 82524, Sohag, Egypt

**Keywords:** Buffaloes, Cystic Ovaries, GnRH/PGF2α, Cytokines, Histopathology

## Abstract

Cystic ovarian disease (COD) with uterine abnormalities is a postpartum reproductive pathology in Egyptian buffaloes causing significant economic losses. In this study, we aimed to employ various diagnostic methods for detecting cystic ovarian disease (COD) in Egyptian buffaloes. tour study assessed the effectiveness of the GnRH/PGF2α protocol as a treatment strategy. Our goal was to determine if this protocol could effectively reduce economic losses associated with cystic ovarian disease and improve herd fertility in Egyptian buffaloes. Eighty Egyptian buffalo cows were included in this study. They were identified to have follicular cysts through rectal examination, which was confirmed by ultrasonography. These buffaloes were then divided into two main groups: the COD Control (untreated) (GA) (n = 40) and COD group (GB) (n = 40) treated with GnRH/PGF2α. According to our immunological studies, buffaloes in the COD-treated group (GB) exhibited significantly lower serum levels of pro-inflammatory cytokines (IL-6, IL-1β, and TNF-α) compared to the control group. This observation was consistent with the decline in E2 levels and the increase in P4 levels (p < 0.01–0.001) observed in the treated animals compared to the untreated group. Furthermore, serum cortisol and glucose concentrations decreased in COD-treated buffaloes. Histopathological examination of ovaries and uterine tissue from slaughtered COD buffaloes has revealed significant structural alterations. These include the presence of ovarian cysts of varying sizes with vacuolar degeneration. Additionally, lymphoplasmacytic endometritis was observed in the uterine tissue of affected animals, featuring degeneration and desquamation of the endometrial lining accompanied by infiltration of mononuclear inflammatory cells. Severe and prolonged cases of COD, which did not respond to treatment, exhibited marked adverse pathological changes upon histopathological assessment of the genital tract. In conclusion**,** hormonal treatment with GnRH/PGF2α appears to be effective in treating COD-affected animals. The study provides valuable insights into the immunological, biochemical, and histopathological aspects of cystic ovaries associated with uterine disorders in Egyptian buffaloes, while also evaluating hormonal treatment for cystic ovarian disease as a means to minimize economic losses and improve herd fertility in this species.

## Introduction

Buffaloes are the world's second-largest milk producer (Bandyopadhyay et al. 2003). Dairy animals, particularly buffaloes, are more susceptible to genital diseases (Ghora 1995). Previous studies (Amin et al. 2021), have indicated that Egyptian buffaloes are known for their inferior reproductive performance. Ovarian dysfunction in buffaloes can impair follicle development and, consequently, estrous cycles. Among the various cysts found on buffalo ovaries, follicular cysts, which account for 25% of all cysts, are the most common (Srinivasan et al. 2017). Cystic ovarian disease (COD) in buffaloes can negatively impact fertility and reproductive performance, thereby affecting the economic parameters of the dairy industry. High milk yield during early lactation can lead to reduced feed intake and lipid mobilization, resulting in negative energy balance and impaired reproduction performance (Diwakar et al. 2017; Baravalle et al. 2015). Gynecological problems such as anestrus, repeat breeding, and cystic ovarian degeneration are common in both cattle and buffaloes, and they can significantly impact reproductive efficiency (Agarwal et al. 2005). The timing of ovarian cyst (OC) formation also influences reproductive performance, with OCs diagnosed after the post-partum having a negative effect on fertility (Gossen and Hoedemaker 2006). Understanding and managing reproductive disorders, including COD, is crucial for maintaining fertility and optimizing economic performance in buffaloes (Abdulkareem et al. 2012).

In large ruminants, cystic ovaries develop from one or more preovulatory follicles larger than 20 mm in diameter that fail to ovulate and persist in the ovary for at least 10 days without a detectable corpus luteum visible on ultrasound (AL-Jabri et al. 2019), thereby interfering with the normal function of the ovary (Murayama et al. 2015). In dairy cattle, ovarian cysts are typically observed early in the postpartum period (Vanholder et al. 2006). At this stage, the animals can manifest clinical signs including, irregular estrus, vigorous Buller cow behavior or persistent estrus, and persistent anestrus (Wiltbank et al. 2002).

The development of cystic ovarian disease depends on a complex interplay of hormones and chemical mediators, which are released under the control of the hypothalamic-pituitary–gonadal axis (Marelli et al. 2014). The formation of cysts on the dominant follicle is a result of hypothalamic-pituitary disorder, which disrupts the preovulatory LH surge (Yoshioka et al. 1996; Hajam et al. 2023). This alteration can have multifactorial origins, involving genetic, phenotypic, and environmental factors (Peter 2004). According to previous studies (Richards and Pangas 2010), the ovulatory process is thought to be a sequence of inflammatory events mediated by cytokines. As a result, they contributed to the development of some ovarian diseases, such as COD (Baravalle et al. 2015).

In addition to the factors listed above, it has also been demonstrated that the hypothalamus may be directly affected by Adrenocorticotropic hormone (ACTH) and cortisol, or indirectly affected by them (Dobson et al. 2000), by causing abnormal circulating progesterone concentrations. The indirect effect is typically associated with stress, leading to an increased level of ACTH and cortisol secretion(Turner et al. 2023), which contributes to the suppression of the hypothalamic-pituitary–ovarian axis activity and the reproductive function (Chen et al. 2022).

Accordingly, several treatment strategies have been used to resolve the cystic condition. These include manual rupture, dexamethasone, progesterone, GnRH, hcG, and PGF2α (Noseir 2015). The GnRH/hCG with PGF2α protocol has been employed in the treatment of follicular cysts to shorten the induced luteal phase (Lamb et al. 2010). Therefore, in this study, we aimed to investigate different diagnostic methods for treating cystic ovarian disease in Egyptian buffaloes and to evaluate the efficacy of the GnRH/PGF2α protocol (day 0 GnRH/PGF, day 14 PGF) in our treatment approach. This approach aims to minimize the economic losses and improve herd fertility in this species.

## Material and methods

### Animals

The study included 80 Egyptian buffalo cows (Bos bubalis). All buffaloes presented at the clinic with a history of irregular estrous cycles and disturbed estrus patterns, and diagnosed with cystic ovarian disease (COD) per rectal examination, were included in the study. These buffaloes, aged between 3 and 7 years, were high milk yielders with a parity ranging from 2 to 4 years. These buffaloes were investigated from December 2020 to September 2022 in dairy farms located in Sohag Governorate, Egypt.

### Study design

The study was carried out in four phases: Animal examination, diagnosis, and grouping (phase I); Treatment strategy (phase II); Collection of Blood for biochemical assay (phase III); tissue samples & histopathological examination (phase IV).

### *Phase I (n* = *80): animal examination, diagnosis, and grouping*

Eighty buffaloes were selected from the Egyptian farms with a history of postpartum anestrum, as they do not show any signs of estrus from the last calving, and some cases persisted anestrum for 4–6 months (days open > 120 days). In this phase, buffalo groups underwent routine reproductive examination by rectal palpation, and ultrasonography, to confirm the health status of the ovary and uterus, twice examination at week or 10-day intervals. These animals were divided into two groups, Group A (COD untreated) (n = 40), and Group B (COD treated) (n = 40), treated with GnRH/PGF2α protocol (Rudowska et al. 2015). Clinical signs and reproductive history were also evaluated according to Murayama et al. (2015).

### The diagnosis involved rectal palpation, US examination, blood samples, and hormonal analysis.

#### Rectal palpation

The buffaloes were secured in a standing position, and routine rectal palpations of the reproductive tracts were performed. Manual palpation of a big (> 25 mm) fluid-filled structure on the ovary in the absence of a corpus luteum (CL) served as the early indicator of the cyst's presence. Either follicular or luteal cysts were assigned to the groups.

#### Ultrasonography examinations (US examination)

The ultrasound examinations were performed using a real-time linear 7.5 MHz linear transrectal probe transducer (Turner et al. 2023). An ultrasound diagnosis was made based on the presence of a non-echogenic area that was at least 25 mm in diameter and was detected during the multiple planes scanning of the ovary to record all of the ovarian features. The cyst was categorized as luteal (with a non-echogenic antrum and a wall > 3 mm thick) or follicular (with a non-echogenic antrum, with a thin wall) (Turner et al. 2023). All follicles larger than 2 mm in diameter (the scanner probe's lowest limit of detection) were counted, along with their number and interior diameter. On a Sony thermal printer, the ultrasonographic images were recorded.

### Diagnostic examination

#### *Phase I (n* = *80): animal examination and diagnosis of ovarian cystic disease*

Behaviorally the diseased buffaloes exhibited signs of sexual aggression (persistent estrus), relaxation of pelvic ligament, sterility hump, or elevated head of the tail. Both rectal and ultrasonographic examinations revealed the presence of thin-walled, watery cysts, unilateral or bilateral in the ovary of these buffaloes. On rectal palpation, the uterine wall was firm, thickened, enlarged, and edematous with a spongy-like consistency.

**Fig. 1:**
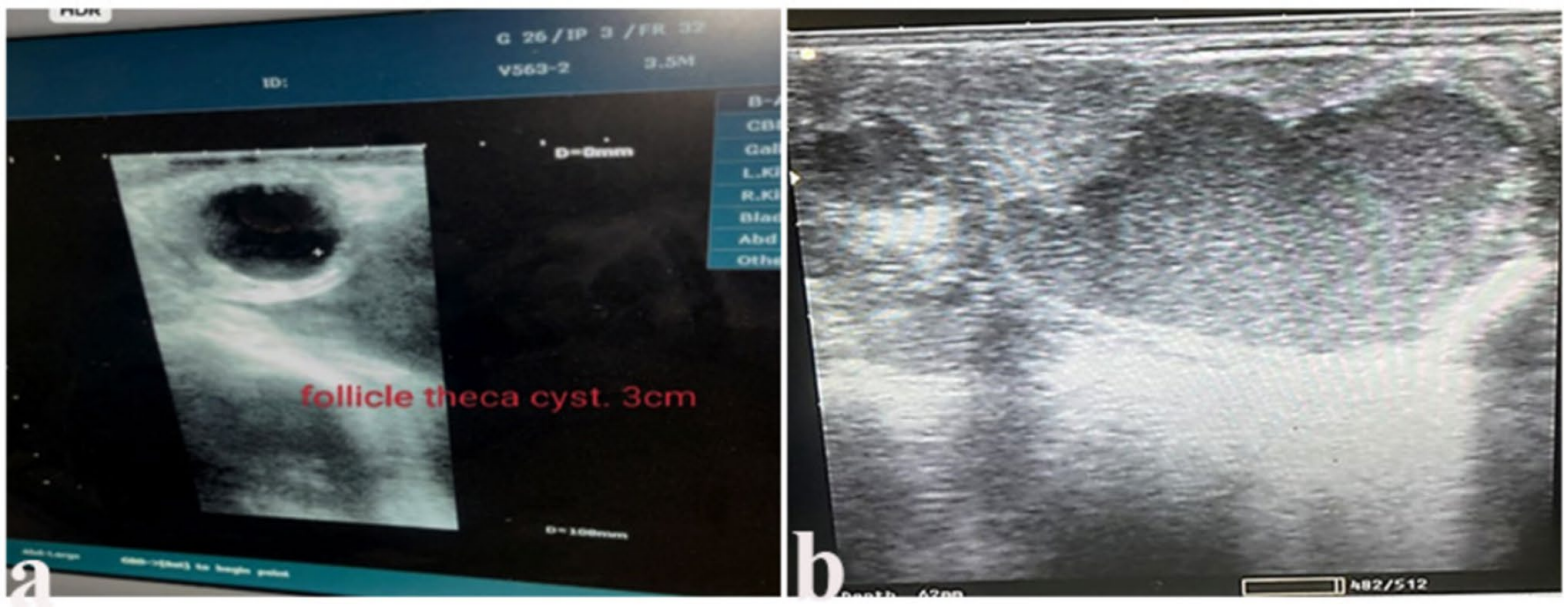
Ultrasonographic graph showing (**A**) COD case showed follicle theca cyst 3cm, (**B**) ovary with multiple follicular cysts

Upon conducting a reproductive ultrasonography examination (Fig. 1), it was confirmed that one or both ovaries lacked luteal tissue, and there were numerous, thin-walled, watery-fluid-filled follicles (≥ 25 mm in diameter) persisting for three consecutive cycles.

### Phase II (n = 40): treatment strategy& trials

#### Treatment protocol

Forty buffaloes, group B (COD treated) (n = 40) were diagnosed with cystic ovary and underwent treatment using the proposed protocol of hormonal treatment, following the steps suggested by Rudowska et al. (2015). The protocol of hormonal treatment was performed step by step as follows: firstl, a dose of 5ml Receptal® VET (GnRH Analogue; intervet, Egypt) was injected intramuscular (IM). Subsequently, a simple follow-up examination via rectal palpation was performed to monitor the luteinization or ovulation of cystic follicles The use of GnRH induces a luteinizing hormone (LH) surge (Souza et al. 2009), resulting in ovulation at 26 to 32 h (Pursley et al. 1995), with most cows ovulating between 28 and 30 h following GnRH administration (Liu et al. 2018). After seven days of Receptal® injection, a dose of 25 mg PGF2α (Lutalyse, Upjohn, Belgium) was injected IM for induction of estrous from luteinized follicles.

### *Phase III (n* = *80): collection of blood for biochemical assay*

#### Blood samples

Blood samples were obtained from the jugular vein of all experimental animals of both groups at the end of the treatment protocol; then centrifuged at 3000 rpm for 20 for serum collection and stored at –20 °C for hormonal and biochemical assay.

#### Biochemical and hormonal analyses

The analysis included estimation of glucose, cholesterol and triglyceride by using a semiautomatic biochemical analyzer and commercially available colorimetric test kits (Biodiagnostic CO, Egypt) calculated according to the manufacturer's instructions.

Determination of estradiol (E2) and progesterone (P4) levels of steroidal reproductive hormones (E2 and P4) were determined using highly specific ELISA Kits with manufacture instructions. Thyroid hormones determination by Enzyme Immunoassay (EIA) was used for the quantitative determination of concentrations of Triiodothyronine (T3), and Thyroxine (T4) in blood serum using commercial test kits according to manufacture instructions. In addition, Cortisol hormone, IL-1β, IL-6 and TNF-αwere determined by ELISA using kits of Cat.no.k7430-100, and MBS269138.

### *Phase VI (n* = *16): tissue samples& histopathological examination*

Buffaloes in group B that were unresponsive to treatment, their owners opted to send them to slaughterhouses. Regarding slaughtering procedures in slaughterhouses, this is done with the knowledge of the competent authorities in accordance with the Egyptian national laws regulating the slaughter process. Samples were collected from slaughtered animals in Egyptian abattoirs in Sohag, where animals were slaughtered by severing the jugular veins using a sharp knife by an experienced person following the Islamic guidelines in Egyptian laws.

The authors' role was limited to collecting samples after slaughter. Samples were collected through the assistance of veterinarians and the slaughterhouse management team.

Tissue samples were obtained from these animals, just after slaughter for histopathological examination. Tissue samples from ovaries and uteri were fixed in 10% neutral buffer formalin for 24 h, and then processed through alcohol series (70%, 90%, 100%) and embedded in paraffin wax. Sections were cut from each block, 5 μm in thickness. Sections were dewaxed with xylene and rehydrated through a descending alcohol series. Slides were stained with Harris hematoxylin and eosin (H & E) (Carleton et al. 1980; Bancroft et al. 1996). All sections were examined and photographed using light microscope OLYMPUS CX43 microscope and photographed with an OLYMPUSDP72 camera adapted to the microscope (Department of Pathology and Clinical Pathology, Faculty of Veterinary Medicine, Sohag University).

### Statistical analysis

The obtained data were analyzed using an independent samples t-test, and one way ANOVA test were used to obtain the difference between mean values of the different groups and the results were confirmed using one way ANOVA test Post Hoc Tukey test by the aid of the SPSS statistical package v22.0 for Windows (IBM, Armonk, NY, USA), the values with different superscript letters in a column are significantly different (p < 0.05).

## Results

Eighty buffaloes were diagnosed with ovarian cystic disease via rectal examination and confirmed by ultrasonographic examinations. Both examinations revealed the presence of thin-walled, watery cysts, unilateral or bilateral in the ovaries of these buffaloes. On rectal palpation, the uterine wall was firm, thickened, enlarged, and edematous with a spongy-like consistency, with endometrial discharges varying from thin watery to mucopurulent or purulent uterine discharge in the affected cases. Behaviorally, the diseased buffaloes were very aggressive sexually (persistent estrus), with relaxed pelvic ligament, sterility hump, or elevated head of the tail.

60% (24 animals) of buffaloes affected with COD responded to the hormonal treatment protocol. The fertile, normal estrus was detected within 15–30 days from treatment which is greatly delayed in the non-treated group (reached up to 120 days). Conversely, the remaining 40% (16 animals) of buffaloes, no estrus was detected.

### Serum immunological assessment

**Fig. 2:**
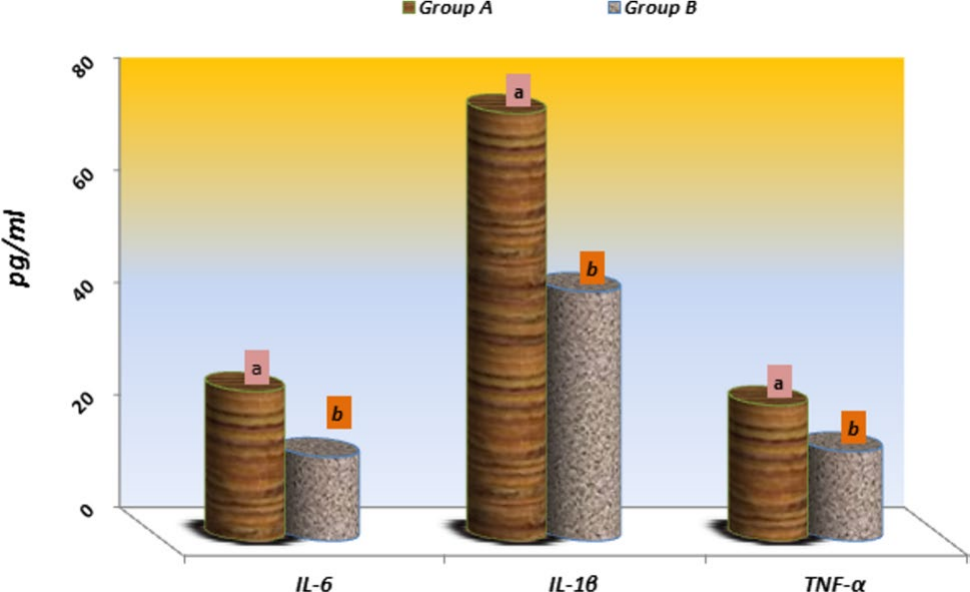
Means of interlukein-6 (IL-6), interlukein-1 (IL-1β) and tumor necrosis factor-α (TNF-α) concentrations in serum of Group A (COD untreated) and Group B (COD treated) of buffaloes. Values are Significance at (*P* < 0.05- 0.01)

The mean expression of pro-inflammatory cytokines interleukin 6 and interleukin 1β (IL-6, IL-1β) and tumor necrosis factor-α (TNF-α) is presented in Fig. 2. It reveals a significant increase (P < 0.01–0.001) in their serum levels in buffaloes with cystic ovaries (Group A) compared to the treated group of buffaloes (Group B) (IL-6: 26.62 ± 1.47 versus 14.94 ± 5.44; IL-1β: 76.09 ± 11.51 versus 44.33 ± 7.88 and TNF- α: 24.12 ± 3.11 versus 15.93 ± 1.54; respectively) (Fig. 2).

### Hormonal assessment

The mean values of P4, E2, cortisol, triiodothyronine (T3), and thyroxin (T4) concentrations in the serum of Group A (COD untreated) and Group B (COD treated) buffaloes were enumerated in Table 1. It indicates a significant higher (P < 0.01–001) serum E2 level in group A than in group B buffaloes. On the other side, P4 levels were significantly increased (P < 0.01–001) in group B compared with those in group A.

**Table 1:** Means of Progesterone (P4) and Estrogen (E2) concentration in serum of Group A (COD untreated) and Group B (COD treated) of buffaloes

Hormones	Group A (COD untreated)	Group B (COD treated)
P4 (ng/ml)	0.16 ± 0.03^a^	0.32 ± 0.81^b^
E2 (pg/ml)	149.58 ± 22.43^a^	69.43 ± 9.09^b^
Cortisol (μg/dl)	102.34^a^	66.82^b^
T3 (ng/ml)	1.47^a^	2.83^b^
T4 (μg/ml)	3.94^a^	7.44^b^

Mean values at the same row marked with different letter were differ at *P* < 0.001

The mean values (± SE) of serum cortisol level were significantly high (p < 0.01) in cystic buffaloes in group A as compared with treated animals in group B (102.34 ± 5.57 versus 66.82 ± 2.17 μg/dl; respectively). However, the significantly lower values (p < 0.01–0.001) of T3 and T4 were observed in COD untreated buffaloes in group A than that in group B treated buffaloes (T3: 1.47 ± 0.21 versus 2.83 ± 0.17; T4: 3.94 ± 0.27 versus 7.44 ± 0.19; respectively).

### Serum biochemical results

**Fig. 3:**
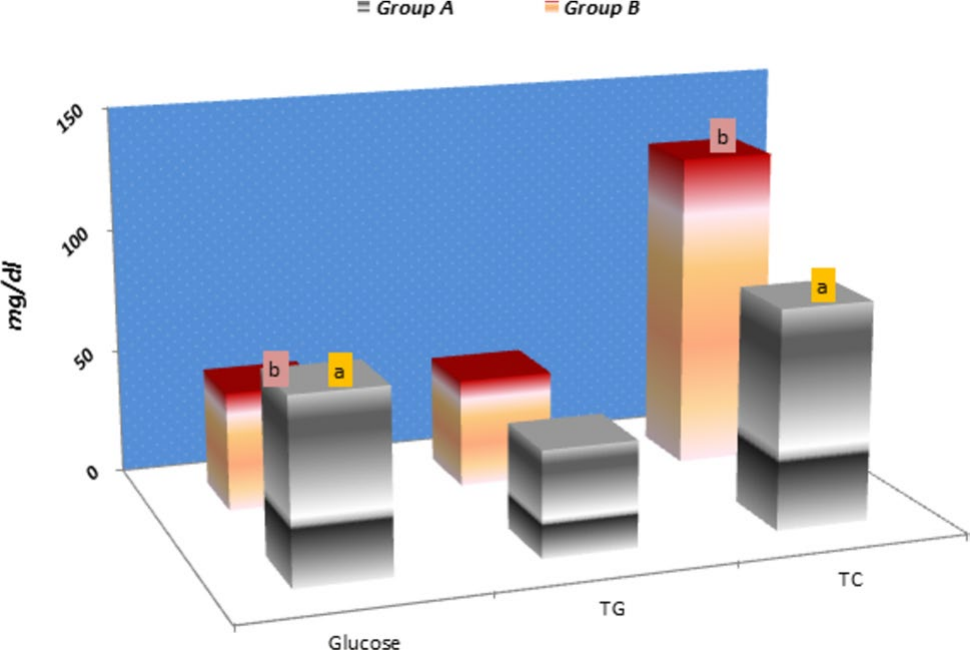
Means of Glucose, total triglyceride (TG) and total cholesterol (TC) concentrations in serum of Group A (COD untreated) and Group B (COD treated) of buffaloes. Values are Significance at (*P* < 0.01)

As the data presented in Fig. 3 the mean values of Glucose, triglyceride (TG) and total cholesterol (TC) concentration in serum of experimental groups. It denoted that a significant increase (p < 0.05) of serum glucose concentration (72.41 ± 6.16 versus 47.46 ± 0.74) of cystic buffaloes in group A than treated (group B). However, the treated group B had a significant higher value of TC (p < 0.01–0.001) than in group A (126.62 ± 4.02 versus 87.00 ± 1.53 mg/dl; respectively). Nevertheless, TG shows no significant differences between the two experimental groups (43.45 ± 2.26 versus 42.01 ± 4.58 mg/dl; respectively).

### Histopathological assessment

#### Gross pathological examination

**Fig. 4:**
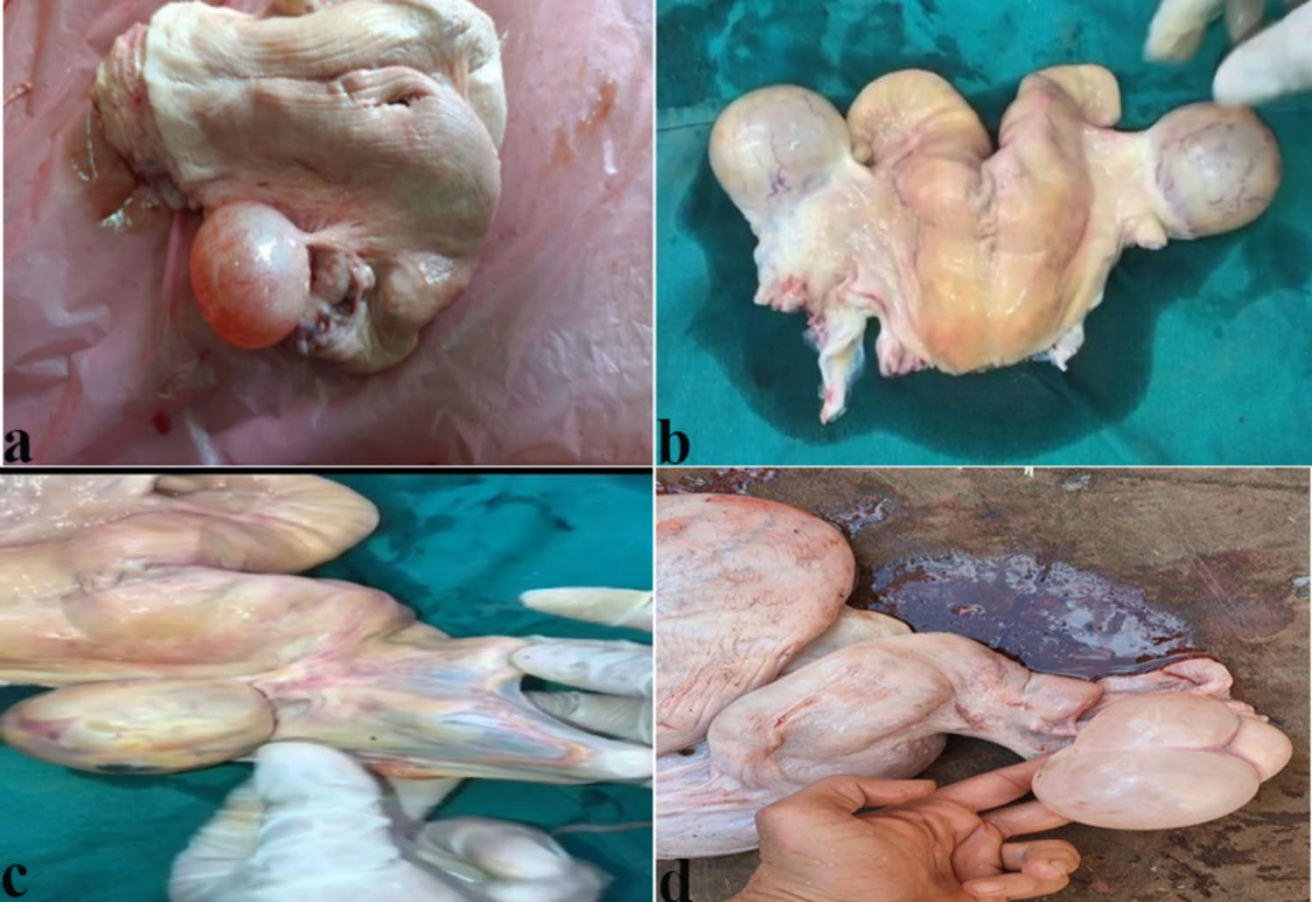
Photograph showing gross morphological changes observed in uterus of slaughtered animals: **a** Unilateral ovarian cyst, **b** Bilateral ovarian cyst, **c** Inflamed uterine tube, **d** Enlarged and inflamed uterus

Tissue samples obtained from group B after slaughtering revealed the presence of large-size, thin-walled ovarian cysts on one or both ovaries. Inflamed, enlarged fallopian tube (F.T.). edematous, swollen, and hyperemic uterus (In some instances, the uterine lumen shows heightened production of thin, serous (watery), or mucoid discharge. while, in other affected cases, it displays thick purulent or mucopurulent discharge. All depend on the degree of uterine pathology (Fig. 4).

#### Microscopical examination

##### Ovaries

**Fig. 5:**
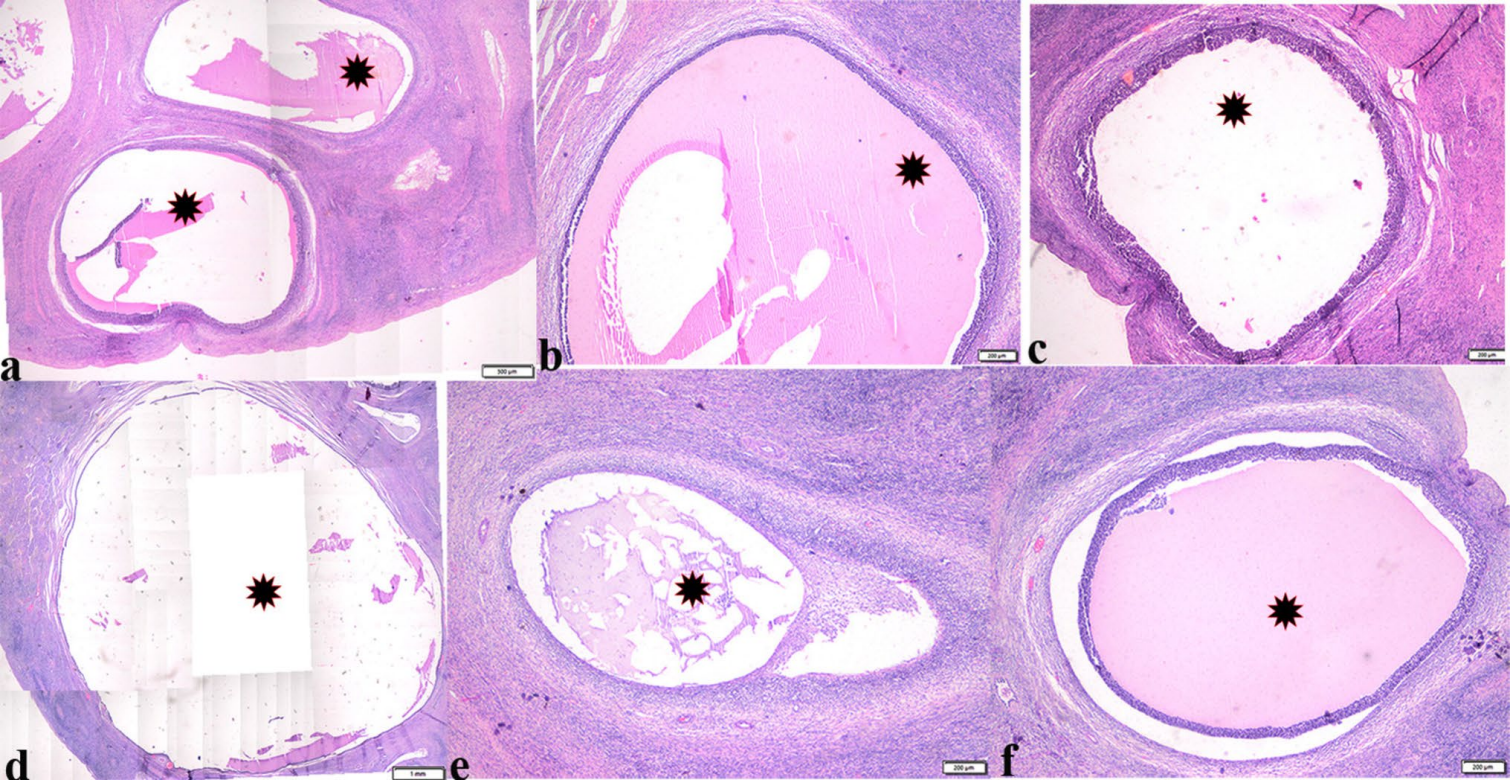
Photomicrograph of ovarian tissue sections from Egyptian buffaloes, showing: Variable sized follicular ovarian cyst (Stars), H&E stain. The bar size was indicated under pictures

**Fig. 6:**
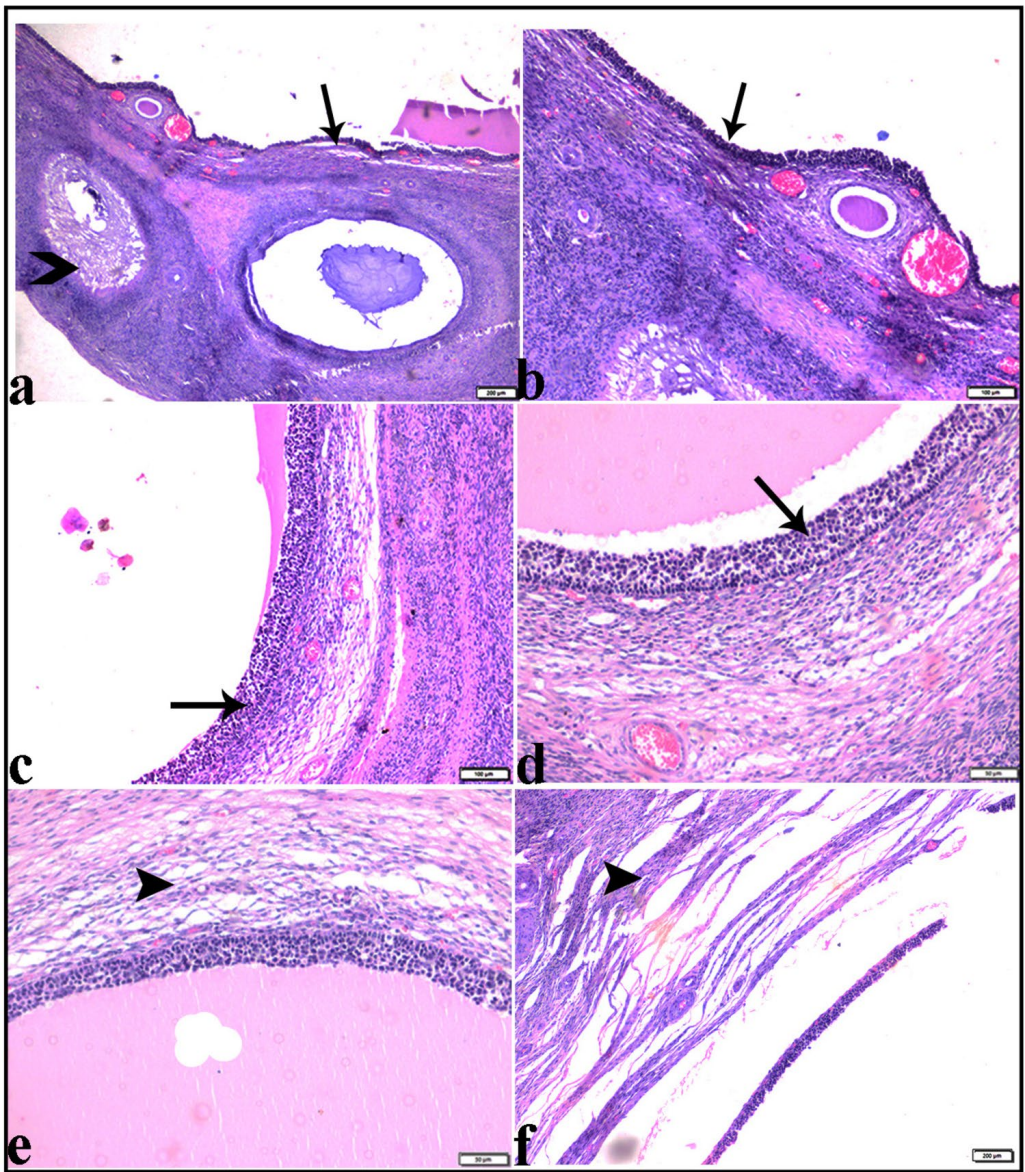
Photomicrograph of ovarian tissue section from Egyptian buffaloes showing: Atretic ovarian follicle (**a**, arrowhead). **b**-**d** Ovarian follicular cysts with thin wall and filled with pale acidophilic residue or blood (arrows), some larger cysts may be lined by a single layer of flattened cells resting on a fibrous capsule (**e** & **f**, arrowheads). H&E stain. The bar size was indicated under pictures

**Fig. 7:**
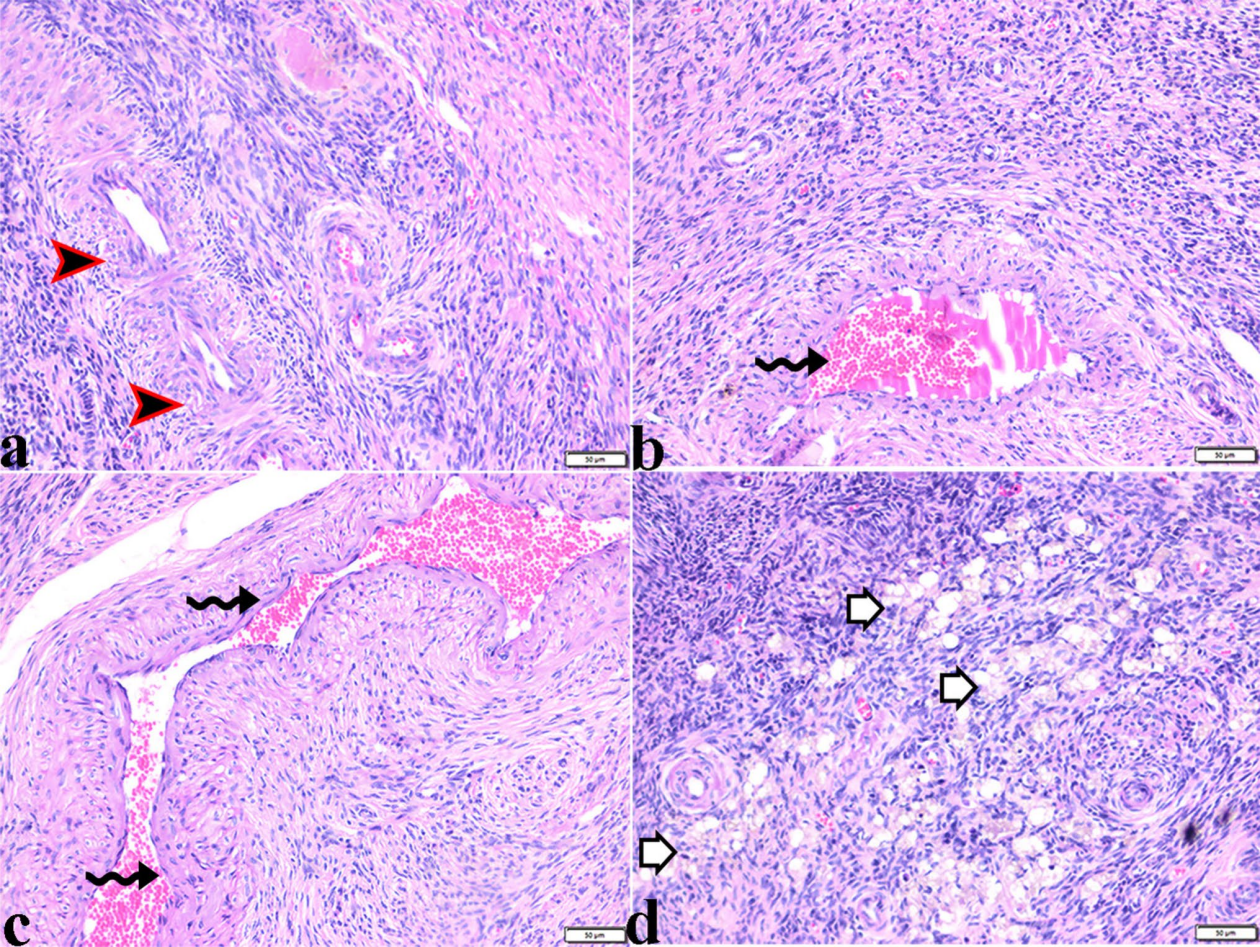
Photomicrograph of ovarian tissue section from Egyptian buffaloes showing: **a** thickening in the vascular wall (arrowheads). **b** & **c** sever vascular dilatation and congestion (zigzag arrows). **d** vacuolar fatty degeneration in the ovarian tissue (white arrows). H&E stain. The bar size was indicated under pictures

Variable-sized follicular ovarian cysts (Fig. 5), some of them were atretic (Fig. 6). The histologic structure of ovarian follicular cysts showed larger cysts lined by a single layer of flattened cells resting on a thin fibrous capsule and filled with pale acidophilic residue or blood (Fig. 6). The granulosa cells of ovarian cysts had from 2 to 4 layers; the basement membrane was present but interrupted in some areas. Additionally, the theca cells appeared hypertrophied, losing their characteristic arrangement parallel to the basement membrane (Fig. 6). The ovarian tissue showed vacuolar fatty degeneration (Fig. 7). Vascular changes in the ovaries were present in dilatation and congestion of vessels with several thickening in the vascular wall (Fig. 7).

##### Uterine tissue

**Fig. 8:**
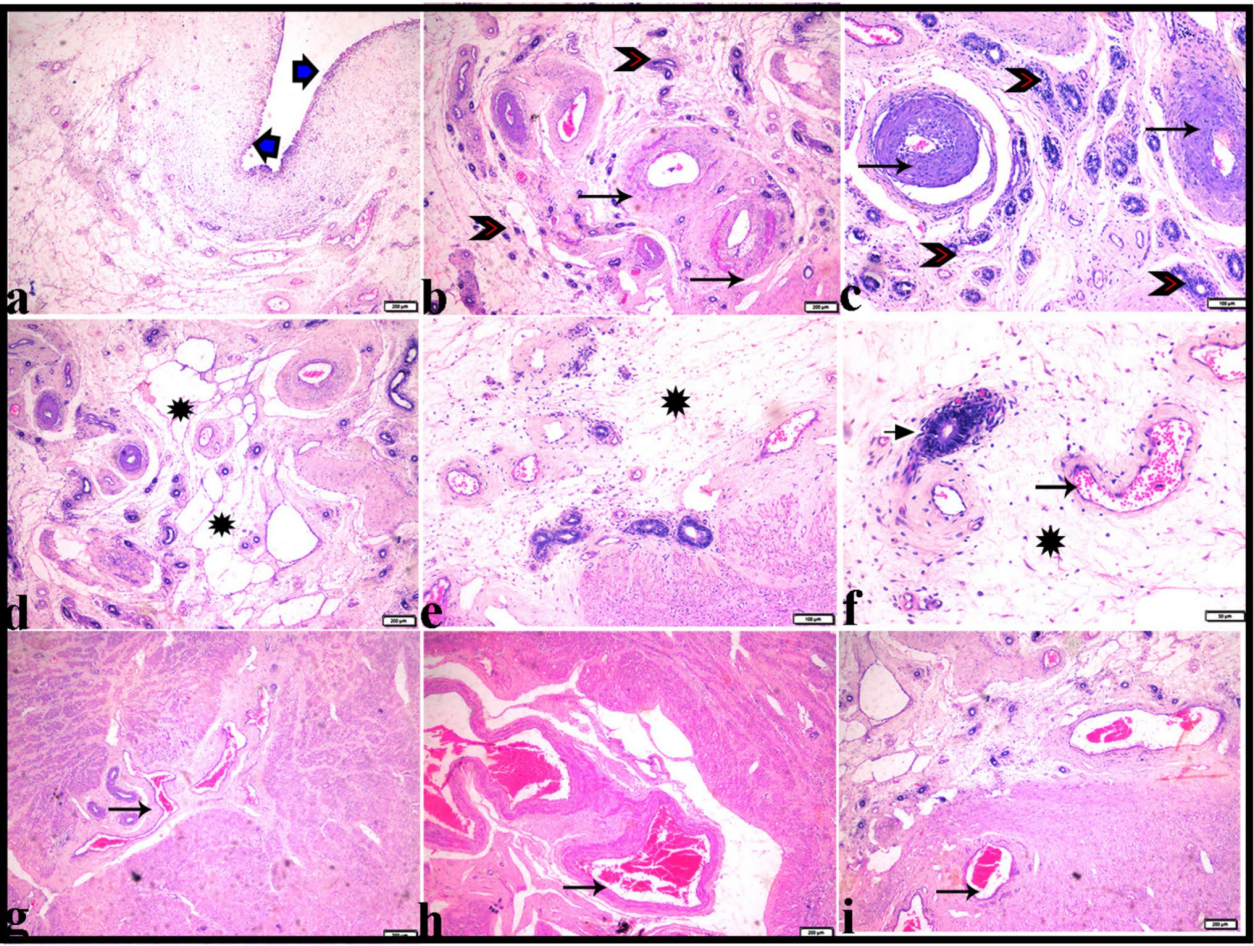
Photomicrograph of uterin tissue section from Egyptian buffaloes showing: sever catarrhal endometritis: **a** degeneration and desquamation of the epithelium lining endometrium (arrowheads). **b** & **c** sever thickening in the vascular wall (arrows), sever atrophy in the endometrial glands (arrowheads). **e–f** catarrhal exudate filled the endometrium (stars) and around the atrophied endometrial glands (arrowhead), mononuclear inflammatory cellular infiltration mostly around the endometrial glands (arrowhead), dilatation and congestion of blood vessel (arrow). **g-f** sever vascular congestion in myometrium (arrows) H&E stain. The bar size was indicated under pictures

**Fig. 9:**
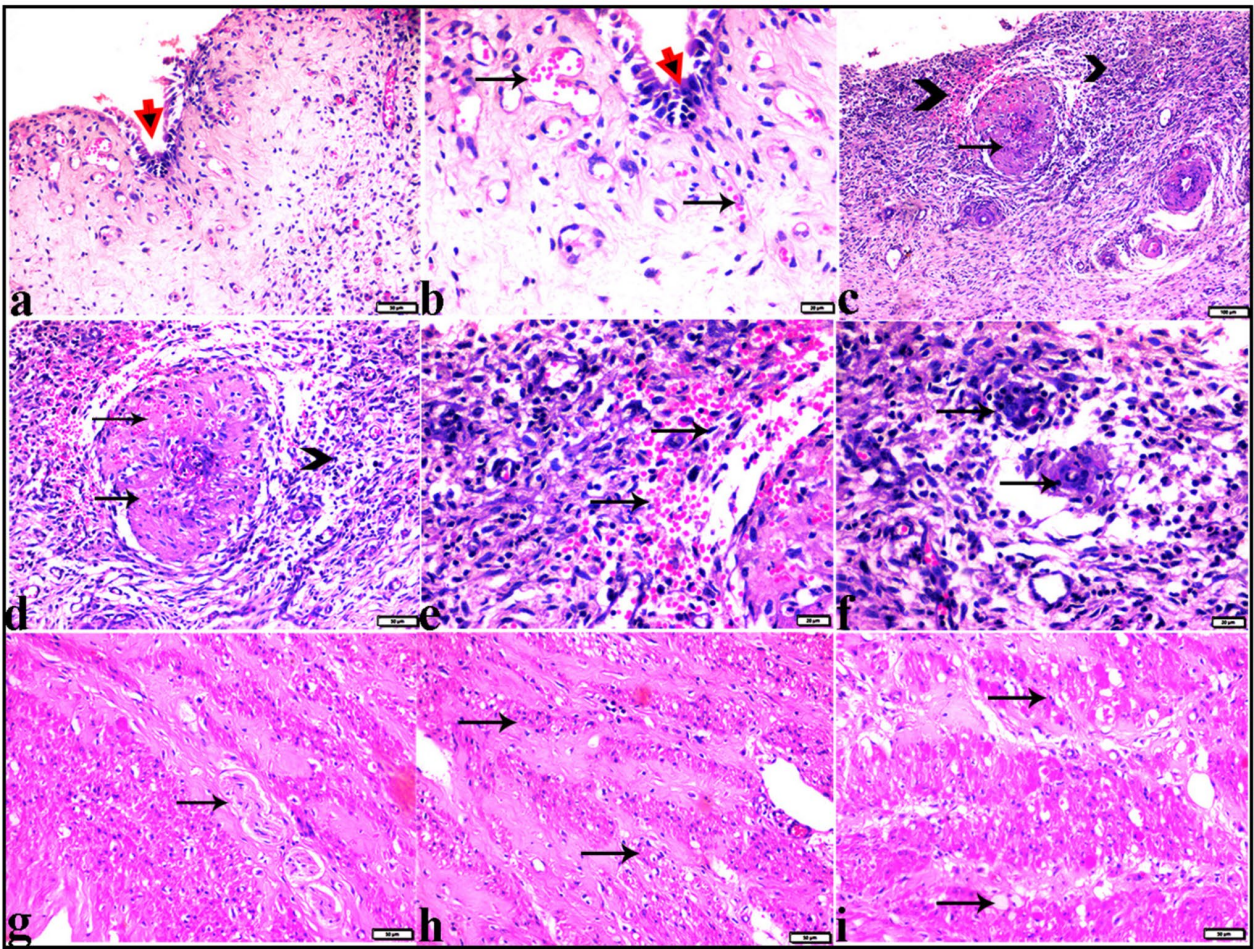
Photomicrograph of uterin tissue section from Egyptian buffaloes showing: sever lymphoplasmocytic endometritis: **a** & **b** degeneration and desquamation of the epithelium lining endometrium (red arrowheads), vascular congestion (arrows). **c** magnified in (**d**) sever degeneration and thickening in vascular wall (thin arrows), marked inflammatory cellular infiltration mainly lymphocytes and plasma cells in endometrium (arrowheads). **e** hemorrhage in endometrium (arrows). **f** sever atrophy in endometrial glands (arrows) with sever periglandular inflammatory cellular infiltration. **g** thickening in vascular wall in myometrium (arrow). **h-i** vacuolar degeneration in myometrium (arrows). H&E stain. The bar size was indicated under pictures

Variable pathological changes were present in the uterine tissue samples including degeneration and desquamation of the lining epithelium of the endometrium with catarrhal exudation filling the endometrium and around the atrophied endometrial glands (Fig. 8). Mononuclear inflammatory cellular infiltration was detected mostly around the endometrial glands. Generalized vascular dilatation was found in most blood vessels with severe vascular congestion in the myometrium region (Fig. 8). Some examined uterine tissue sections showed severe lymphoplasmocytic endometritis, characterized by desquamation of the endometrial epithelium and marked inflammatory cellular infiltration, mainly lymphocytes and plasma cells. Additionally, severe atrophy in endometrial glands was observed, accompanied by severe periglandular inflammatory cellular infiltration (Fig. 9). Vascular changes were characterized by severe degeneration and thickening in the vascular wall (Fig. 9).

## Discussion

Cystic ovaries are considered the most critical ovarian condition in buffalo and occur early after parturition causing a prolonged inter-calving period resulting in infertility and severe economic losses in the industry (Vanholder et al. 2006). It is characterized by the presence of large pre-ovulatory follicles (≥ 25 mm in diameter), thin-walled and filled with a watery fluid, and persists for three successive repeated rectal palpations with the absence of luteal tissue on one or two ovaries as observed in our results. Similar findings were obtained in a previous study (Charoennam et al. 2019)**.** Moreover, our rectal examination revealed an enlarged and edematous uterine wall with a spongy-like consistency, which is consistent with previously recorded findings (Satheshkumar 2022; Hussaini et al. 2022)**.**

Ultrasonography is extensively used for reproductive management and disease diagnosis in domestic animals, including buffaloes (Moonmanee et al. 2017). In Italian Mediterranean Buffaloes, it aids in optimizing reproductive management and diagnosing conditions like ovarian cystic degeneration, hydrosalpinx, salpingitis, metritis, pyometra, and pneumovagina (Acorda 2019). In postpartum Egyptian buffaloes, trans-rectal Doppler sonography effectively evaluates ovarian and uterine hemodynamics during the puerperium (Stelletta et al. 2018). Additionally, a short-term controlled internal drug release (CIDR)-based protocol with prostaglandin F2α (PGF2α) and human chorionic gonadotropin (hCG) injections was found to effectively treat large cystic and inactive ovaries in anestrous Mehsana buffaloes, allowing for subsequent pregnancy (El-Sherbiny et al. 2019). However, these studies did not specifically mention histopathological and serological investigations."

The observed aggressive sexual behavior of affected buffaloes, along with the relaxation of pelvic ligaments in our results, was consistent with findings reported by Vanholder et al. (2006). With regards to our hormonal treatment protocol, follicular cysts in buffalo was successfully treated with a success rate which is similar to that reported in cows (Garverick 1997). Additionally, the prompt restoration of fertility and normal estrus within a short timeframe (15–30 days) following hormonal treatment in COD buffaloes, in contrast to the significantly delayed return observed in the non-treated group (which could extend up to 120 days). This observation highlights the beneficial impact of the GnRH/PGF2α protocol on reproductive tract performance, as indicated by a previous study (Yotov et al. 2014). Also, shortens the interval day to conception (Garverick et al. 1976), consequently decreasing the severe economic loss of dairy farms due to COD.

On the other hand, buffaloes that did not respond to treatment suffered from several pathological conditions of the ovary and uterus, as noted in our histopathological examination.IL-1, IL-6, IL-8, and TNF-α levels were found to be increased in ovarian hyperstimulation syndrome (Kasum 2010). Additionally, an increase in the serum levels of IL-6 and TNF-α was observed in polycystic ovary syndrome (Wu et al. 2004). These studies suggest that alterations in the expression of cytokines are considered key evidence of the early development of cystic ovarian disease (COD) in cows, which may contribute to follicular persistence and ovulation failure found in cattle with follicular cysts (Stassi et al. 2017). Accordingly, cytokines can be used in the diagnosis of the pathological changes occurring in the ovaries (Brodzki et al. 2019).

The significant increase (P < 0.01- 0.001) in serum level of pro-inflammatory cytokines IL-6, IL-1β, and TNF-α in untreated groups in comparison with the treated animals as demonstrated in our study was with the following (Lima et al. 2019) in dairy cows. They suggested these cystic cows had marked metabolically and had an immunological disturbance. In addition, (Baravalle et al. 2015). denoted an increased expression of IL-6 and TNF-α in the fluid of cystic ovaries in the untreated group, providing this may be related to the presence of follicular cysts. Illustrating, the disturbance in pro-inflammatory cytokines expression may be due to ovulation failure which assists in the formation of follicular cysts. Amato et al. (2003) found higher levels of TNF-α and IL-6 in serum in infertile women with polycystic ovaries than in normal. In addition, previous research has denoted higher expression of these proinflammatory cytokines in cystic follicles of cattle, indicating the alteration of these proteins' expression which may be related to the occurrence of follicular cysts (Stassi et al. 2017)**.**

Endometritis diagnosis in the current study was based on histological examination. The present study indicated that buffaloes with cystic ovaries suffered from obviously high E2 and low P4 levels (p < 0.01–0.001) than treated buffaloes, these observations were similar to that reported by (El-Sakkar et al. 2008) in buffalo and (Mohamed and Hussein 2004) in camel. The significantly lower P4 in buffaloes with untreated cystic ovaries in our results were similar to the findings of Tsujii et al. (2016). In the same side (Hamilton et al. 1995) cited that serum of a cystic cow had obvious lower P4 concentration and high basal luteinizing hormone (LH) level than the normal cow. Also, (Douthwaite and Dobson 2000) reported that the serum P4 level was higher in cows with lutein cysts than in cows with follicular cysts, this may be related to lower luteinization of the follicular cyst with decreased P4 production (Zeitoun 2003). The main effect of our hormonal protocol is that Synthetic GnRH stimulates the luteinization of cystic follicles and the formation of the corpus luteum (CL), increasing the concentration of progesterone levels (Mollo et al. 2012). Following by injection of Lutalyse (PGF2α) on the 7th day after GnRH injection is for luteolysis of formed CL, so the dominant follicle of the next follicular wave begins to develop (Nanda et al. 1988). So, our results were in accordance with (Gumen 2003) who reported that a combination of GnRH /PGF2 α is more efficient in the treatment of cysts in dairy cows.

The significant elevation in E2 levels in buffaloes with cystic ovary and endometritis aligns with previous studies (Mabrouk 1989; Homeida et al. 1991) which found a strong correlation between follicular size and E2 level.. Homeida et al. confirmed that E2 concentration was parallel to follicular size (Homeida et al. 1988). The same results were obtained by Hamilton et al. who cited a higher E2 concentration in cows with cystic ovary than ovulatory cycles (Hamilton et al. 1995). In addition, Roberts (1956) also found that cows with polycystic ovaries have higher serum E2, likely due to persistent ovarian follicles with more LH receptors, stimulating follicle androgen production and resulting in increased E2 (Armstrong et al. 2002)**.**

The current study revealed a significant increased serum cortisol level (p < 0.01) in buffaloes with cystic ovary. This result goes along with the findings of (AL-Jabri et al. 2019; Mimoune et al. 2019; 2017)**.** Immediately after parturition, the negative energy balance (NEB), poor nutrition, and lactation act as stress on animals which causes increased cortisol which suppresses the LH surge, failure of ovulation, and follicle persistence for prolonged time (Osman et al. 2017).

The current study revealed that the values of the T3 and T4 were significantly lower (p < 0.01–0.001) in buffaloes with cystic ovaries than in treated buffaloes. These findings were in the same line as reported by (Metwelly et al. 2004) who noted in their study on she-camel that the lowest levels of the T3 and T4 were found in cystic ovaries. Additionally, (Shu et al. 2011) found that in hypothyroid cases of developing ovarian cysts, hormone replacement therapy results in a restoration of the euthyroid state as well as a regression of symptoms and ovarian enlargement.

Glucose plays a crucial role in ovarian metabolism and is considered as an energy source for ovarian activity as it's metabolized by the ovary leading to lactate formation through the anaerobic pathways, (Leroy et al. 2004). Our present study observed a significant increase (p < 0.01) in glucose concentration in the serum of cystic buffaloes in comparison to the treated buffaloes were in agreed with (Mimoune et al. 2017) in cattle. This increase may be due to the cystic follicles' capability to filter and restore high levels of glucose from the blood, which is needed for their development (Abd Ellah et al. 2010)**.** In addition, Albomohsen et al. found that with follicular development, the blood-follicular barrier permeability was increased; this may considered another cause for such increase (Albomohsen et al. 2011).

Cholesterol is considered a key precursor for steroid hormones in both males and females (Shang et al. 2019). Follicular fluid (FF), cholesterol binds with high-density lipoprotein (HDL-P), which can pass through the blood-follicle barrier (Leroy et al. 2004). In the present study, the low total cholesterol (TC) levels align with previous findings by Mimoune et al. (2017). This reduction may be due to its utilization in steroid biosynthesis.

Lipids are stored in the form of triglyceride (TG), their hydrolysis gives glycerol molecule and three molecules of fatty acid. Therefore, TG may be considered as another energy source during follicular growth and development (Tabatabaei and Mamoei 2011)**.** In our study, TG showed no significant differences between the serum of cystic ovaries and treated, which agreed with the data of Ali et al. in camels (Ali et al. 2010).

Cystic ovarian disease (COD) is a reproductive illness that primarily affects dairy cattle, but it has also been observed in buffaloes. This illness can cause substantial histological changes in the ovary and uterus, resulting in reproductive dysfunction. Histopathological investigation sheds light on the structural abnormalities associated with COD in various organs.

COD is distinguished in the ovary by the appearance of cystic formations, which might impair normal follicular growth and hormone synthesis. Histopathologically, these cysts show epithelial lining, fluid-filled cavities, and occasionally hemorrhagic material. Studies in buffaloes have shown histological abnormalities such as follicular cysts, and cystic corpus luteum. Our results were in accordance with previous studies on this matter (Purohit 2015; Agarwal et al. 2005).

Furthermore, the histopathological effects of COD extend to the uterus, where hormonal imbalances and ovarian function might affect endometrial shape and functionality, as described elsewhere (Sanjay et al. 2016). Overall, histological study of the ovary and uterine gives useful information about the pathophysiology of cystic ovarian illness in buffaloes. Understanding these histological changes is critical for accurately detecting and controlling COD in buffalo herds to maximize reproductive output.

Ultimately, the various biochemical and hormonal changes recorded in our study were consistent with the histopathological findings observed in group B animals. Our results align with previous findings (Braw-Tal et al. 2009; Nora et al. 2018).

## Conclusion

COD significantly impacts animal fertility. Hormonal protocol treatment with GnRH/PGF2α appears to be more efficient in tackling the problem of ovarian cysts in buffaloes. This is evidenced by increased progesterone levels, notable improvements in other serum biochemical profiles, and enhanced estrus observed in treated cystic animals. While marked adverse pathological changes on serological assessment and genital tract which were observed in cystic buffaloes which did not respond to treatment be attributed to the persistence of cysts over an extended period.

The various biochemical and hormonal changes recorded in our study were consistent with the histopathological findings observed in group B animals, aligning with previous research. Hormonal treatments, such as GnRH/PGF2α therapy, can help restore reproductive function in buffaloes with ovarian cysts. Monitoring progesterone levels and serum biochemical profiles is useful for evaluating the effectiveness of the therapy and the overall health of the animals. Additionally, further research into alternative approaches, such as antioxidant supplementation combined with GnRH/PGF2α therapy, could provide insights into new or complementary treatments for reducing ovarian cysts and enhancing female reproductive efficiency.

Overall, this excerpt highlights the importance of continued research and a diverse strategy for managing ovarian cysts and improving reproductive outcomes in buffaloes, ultimately contributing to the long-term viability and productivity of buffalo production systems.

## Data Availability

The data supporting this study’s findings are available on request from the corresponding author.

## Abbreviations

COD: Cystic ovarian disease
IL: Interleukin
TNF-α: Tumor necrosis factor-α
OC: Ovarian cyst
ACTH: Adrenocorticotropic hormone
CL: Corpus luteum
T3: Triiodothyronine
T4: Thyroxin
P4: Progesterone
E2: Estrogen
PGF2α: Prostaglandin F2α
hCG: Human chorionic gonadotropin
FF: Follicular fluid
TG: Triglyceride
TC: Total cholesterol
